# Nonlinear photomechanics of nematic networks: upscaling microscopic behaviour to macroscopic deformation

**DOI:** 10.1038/srep20026

**Published:** 2016-02-01

**Authors:** Hayoung Chung, Joonmyung Choi, Jung-Hoon Yun, Maenghyo Cho

**Affiliations:** 1 School of Mechanical and Aerospace Engineering, Seoul National University, Seoul, 151-744, South Korea

## Abstract

A liquid crystal network whose chromophores are functionalized by photochromic dye exhibits light-induced mechanical behaviour. As a result, the micro-scaled thermotropic traits of the network and the macroscopic phase behaviour are both influenced as light alternates the shape of the dyes. In this paper, we present an analysis of this photomechanical behaviour based on the proposed multiscale framework, which incorporates the molecular details of microstate evolution into a continuum-based understanding. The effects of *trans-to-cis* photoisomerization driven by actinic light irradiation are first examined using molecular dynamics simulations, and are compared against the predictions of the classical dilution model; this reveals certain characteristics of mesogenic interaction upon isomerization, followed by changes in the polymeric structure. We then upscale the thermotropic phase-related information with the aid of a nonlinear finite element analysis; macroscopic deflection with respect to the wide ranges of temperature and actinic light intensity are thereby examined, which reveals that the classical model underestimates the true deformation. This work therefore provides measures for analysing photomechanics in general by bridging the gap between the micro- and macro-scales.

Polymeric chains that are crosslinked with rigid photochromic chromophores – known as photo-responsive polymer networks (PRPNs) – change their shape (from the macroscopic perspective) upon light irradiation^1^,^2^,^3^. Photoisomerization contributes to such opto-mechanical coupling. The process of *trans-to-cis* isomerization (driven by actinic light irradiation) perturbs the original symmetry; on the other hand, *cis-to-trans* isomerization, which is due to either increased temperature or visible light irradiation, accomplishes the inverse^3^,^4^,^5^. This photomechanical process, which can be driven by remote and wireless stimuli, has been envisaged as a novel opto-mechanical mechanism^6^,^7^,^8^. Our current understanding of the physical factors at play, however, relies heavily on experimental measures^3^,^4^,^5^,^9^,^10^,^11^, as the comprehensive theoretical approach requires a comprehensive knowledge of broad, interdisciplinary physical regimes that range from molecular geometry (i.e., *cis-* and *trans-* shapes) to manipulating macroscopic deformations.

Concerning deconvoluted photomechanics, on the other hand, the existing theoretical approaches have been successful in describing each photomechanical behaviour from the perspective of different physical regimes of interest. For example, a polymeric description of such micropolar materials based on continuum mechanics has succeeded in reproducing certain details concerning the anomalous behaviour of nematic solids: e.g. the non-convexity of the energy landscape^12^,^13^,^14^, the dynamics of stress evolution and diverse stress-accommodating shape change (such as bent elastica^15^,^16^,^17^,^18^,^19^,^20^,^21^), etc. At the other side of the analysis, small-scale simulations^22^,^23^,^24^,^25^ have demonstrated that thermomechanical phase phenomena are related to the light-induced effects, and that they correlate well with the observed light-induced motions. Although a few recent studies have suggested connections between changes in nematic order and large-scale behaviour^26^,^27^,^28^,^29^,^30^,^31^, these works still resort to classical modelling by relying on Landau-de Gennes coupling along with the linear dilute model. Accordingly, such approaches fail to take into account crucial molecular details regarding light irradiation.

In this article, we pave the way toward reducing the knowledge gap between the two levels (micro and macro) in order to simulate the large-scale behaviour of PRPNs in terms of *cis-trans* isomerization. More specifically, we examine the azobenzene-based acrylate side-chain PRPNs^9^,^32^,^33^,^34^,^35^,^36^,^37^,^38^, the backbones of which are composed of acrylate monomers and crosslinkers. Such glassy networks exhibit various light-induced bending behaviours, which result from the modulation of certain physical properties (e.g. Kuhn length, crosslinking density). Their in-plane shrinkage is overshadowed by out-of-plane deflection or bending, because the *trans-to-cis* isomerization processes of PRPNs are likely to induce in-plane mechanical strain caused by the high modulus of the structure.

First, the small-scale behaviour of the structure is examined via molecular dynamics (MD) simulations, whereby an increased number of *cis-* molecules influences the thermotropic properties that result from light-induced *trans-to-cis* isomerization. We examine a heuristic equation^39^ that substitutes Landau expansion for Maier-Saupe phase transition^40^ and modify it parametrically in order to properly consider the influence of kinked *cis-* molecules. These thermotropic parameters reflect detailed molecular effects that are not captured by experimental observations; the parameters are extracted by averaging across wide windows of both time and temperature. The MD simulations used herein are largely indebted to recent work^23^ regarding how an increase in the *cis-* population is correlated with photo-induced cell shrinkage and order collapse in general; however, this macroscopic viewpoint fails to grasp key microscopic information, as was evident in the case of the experimental approach. We therefore focus on extracting the modulated thermomechanical phase behaviours, which reflect changes in the long-range mesogenic interactions in the presence of short-range atomic interactions.

Next, we present the sequential multiscale framework in which microstate-related parameters become upscaled and related to macroscopic deformations. The *in silico* experimentation reveals that microstate behaviour relates non-linearly to the increased number of *cis-* molecules, and the mechanical properties (such as residual stress) are computed accordingly. Driven by the stress gradient that evolves within the structure, the bending behaviour is therefore examined and discussed in the context of various external conditions. As a result, the predictions of the multiscale framework are significantly different from those of the classical dilution model^1^,^2^,^39^, wherein the isotropic-nematic temperature barrier is assumed to decrease linearly with the increasing number of isomers. Above all, polymeric configurations are freely modifiable since numerical simulations rather than experimental investigations constitute the basis of the framework. The present methodology therefore provides valuable insight toward understanding and even predicting the photomechanical nature of arbitrary PRPN structures.

## Results and Discussion

### Thermomechanical phase behaviour of PRPNs

**Figure 1:**
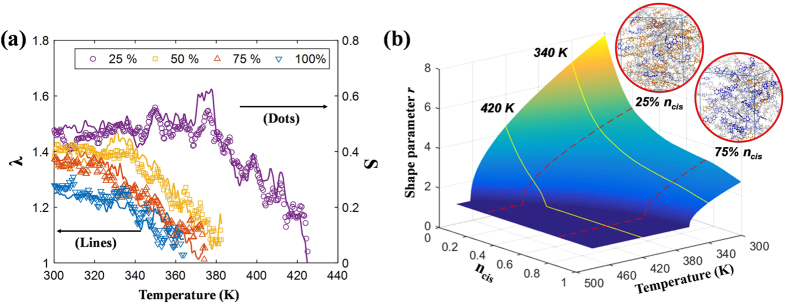
(**a**) Variation of the shrinkage parameter *λ* and order parameter *S* during temperature elevation with different photoisomerization ratios. (**b**) Shape parameter *r* according to various temperatures and *cis-* populations 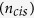; iso-temperature curve (solid) and iso-*cis-*population curve (dotted) to illustrate nonlinearity. Inset figures illustrate 25%-isomerized (upper) and 100%-isomerized (lower) unit cells used in the molecular dynamics (MD) simulation. *Cis-*molecules are blue, *trans-*molecules are brown, and hydrocarbons are grey.

Figure 1 illustrates the MD simulation results of microscopic changes induced by light irradiation, as well as thermal stimuli. (See Methods for detailed descriptions of conditions such as cell generation, atomic interaction, etc.) The mesogenic portions and orientations of each PRPN unit cell are parameterized by the orientational order *S* and cell shrinkage parameter *λ*. *S* is the ensemble average of the orientation, whereas *λ* indicates the fractional change in the unit cell’s length *L* along the direction of initial alignment. (Detailed computations can be found in Methods.) Above all, we observe a salient linearity between *S* and *λ*^41^, which demonstrates a strong interaction between the optical order and backbone conformation. The relationship between these two parameters is summarized in Eq. (1), wherein a nematic order-polymeric backbone coupling parameter *α* describes the sensitivity of the polymeric structure to the perturbed rotational symmetry. The phase change of the mesogenic molecules is also of interest. In contrast to the behaviour associated with the ideal Maier-Saupe phase transition model of liquid crystals^40^, the PRPN cell exhibits a roughly 2^nd^-order phase transition^22^,^39^,^42^, as shown in Eq. (2). The orientational order-temperature (*S–T*) curves are parameterized by the order-clearing temperature *T*_*c*_ and the critical exponent ζ, as shown in Eq. (2).


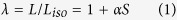



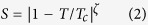


Table 1 lists the MD-derived parameters with different populations of *cis-* molecules. In contrast to the classical dilute model, wherein all parameters but *T*_*c*_ remain constant with light irradiation, all the listed thermotropic parameters (i.e. *T*_*c*_, *ζ*, *α*) vary monotonically upon the introduction of kinked molecules.

These trends clearly demonstrate two possible effects that may result from a change in the interactions between mesogens as the concentration of *cis-* molecules increases. Figure 1(b) illustrates the change in the shape parameter *r* of a PRPN as a function of the *cis-* population and the temperature. The shape parameter indicates the length ratio between the longitudinal and transverse directions of the polymeric shape in the nematic case; the fact that it decreases as the *cis-* population increases suggests that the anisotropy of the polymeric structure decreases. As assumed in the classical dilute model, wherein photoisomerization decreases the number of molecules involved in uniaxial order, the increased number of *cis-* isomers lowers the thermal barrier required to clear the uniaxial distribution of mesogens.

Additional effects that stem from the polymer conformation characteristics can also be found in the present acrylate-based model. For instance, crosslinked mesogenic molecules are densely packed, and thus their long-range interactions are easily perturbed by geometrical changes in the constituents. Newly introduced kinked molecules perturb the relaxed orientation of neighbouring rigid molecules, causing them to deviate from their initial alignment. As a result, this loosens the stacking of the rigid mesogens (i.e. the distance between them is lengthened and their orientations deviate from one from another), and the strength of the interaction between rigid rods therefore decreases. This modification of long-range interactions not only reduces the orientational order, but also renders the polymeric structures less responsive to the decreasing order (lower *α*). As a result, their phase transition behaviour becomes less abrupt near the clearing temperature (higher *ζ*). This represents a further deviation from the ideal 1^st^-order phase transition as the stacking effect becomes diminished. Additionally, comparing the thermotropic parameters (*α*, *ζ*) with those of elastomer-based PRPNs^39^ demonstrates the aforementioned differences that result from stacking characteristics.

This line of thought, however, is not a complete description of the observed phenomena. The retained thermomechanical phase behaviour when 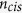
= 1, for example, also contradicts the basis of the dilute model that expects fully photo-isomerized cells lose their long-range interactions. Such inconsistency in the classical viewpoint may be due to auxiliary effects that possibly stem from various and unaccounted origins, not to mention the aforementioned dense stacking effect. For instance, the change of the molecular architecture from side- to main-chain^3^,^4^,^5^ that drives the change of the photomechanical properties exemplifies such an effect; the kinked dyes (i.e., *cis-*chromophore) contribute differently to the mesogenic order. Furthermore, the MD simulation results have shown that local voids and intrinsic stresses often evolve near the area where *trans-to-cis* isomerization occurs as the atomic positions are translated. These auxiliary effects and their influence on the phase behaviours are to be discussed in future work.

### Multiscale simulation of light-induced bending

**Figure 2:**
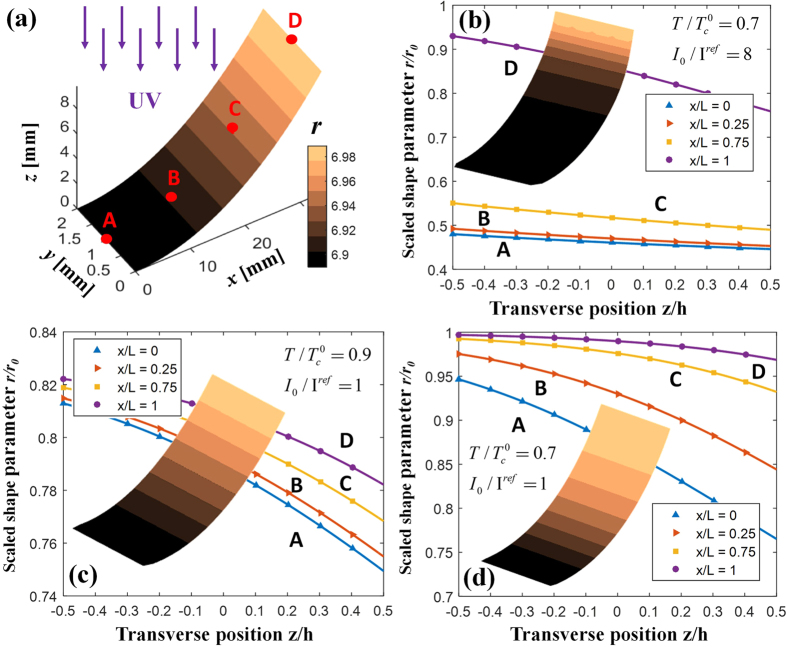
Photo-responsive polymer network (PRPN) bending induced by light travelling in the –z direction. Colouring indicates the shape parameter *r* of the upper surface. For equally spaced positions (i.e. **a**–**d**), the transverse position vs. normalized shape parameters of different intensities 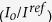 and temperatures 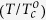 is plotted. (h represents the thickness of the strip).

With the aid of the multiscale-based framework, we investigate the bending behaviours by which a PRPN deforms under actinic light^32^,^33^,^34^,^35^,^36^,^37^. As shown in Fig. 2(a), a cantilevered thin PRPN strip is bombarded with light (intensity: *I*_*o*_) that travels in the −z direction, as well as a separate stimulus for elevating the temperature *T*; these external stimuli drive the *trans-to-cis* isomerization and the thermal-induced *cis-to-trans* isomerization, respectively. Herein the deformations related to light and temperature are discussed in a scaled manner by introducing the reference light intensity 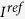 and time constant 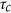. The initial director 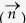 is aligned along the x-axis, so the PRPN’s deflections and rotations are each defined by the nodal displacement in the z direction and rotation of the normal vector along the y-axis, as the significant bending curvature change is observed in the x-z plane. A deep nematic phase is assumed to exist prior to and during crosslinking. We refer to the initial temperature as 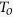, which is lower than the clearing temperature 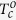 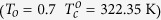 under non-irradiated conditions; the initial polymer structure is therefore highly anisotropic (i.e. shape parameter *r* > 1).

The results presented in Fig. 2 illustrate the deformation of the strip (colour indicates the value of *r*) as well as the varying shape parameter along the transverse z direction found at points A, B, C, and D, which are plotted along the x-axis; in this context, h represents the thickness of the strip. These results indicate that the bending behaviour is strongly affected by varying the stimuli. Typical bending occurs where the surface-dominant shape parameter change is observed under weak light conditions, which follow Beer’s law. For intense light conditions 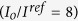, on the other hand, light penetrates deeply into the material, which is known as the photo-bleaching effect, and thus the gradient of the shape parameter is reduced; 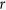 converges to unity (i.e. the polymeric shape becomes isotropic and thus shrinks in the longitudinal direction) near the base of the PRPN cantilever as a large number of *cis*- molecules are stacked, which reduces 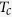 closer to the operating temperature. In this way, in-plane shrinkage overshadows the bending moment. Elevating the temperature 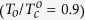 significantly enhances the backward reaction of isomerization and provides polymeric flexibility, thereby reducing deformation.

**Figure 3:**
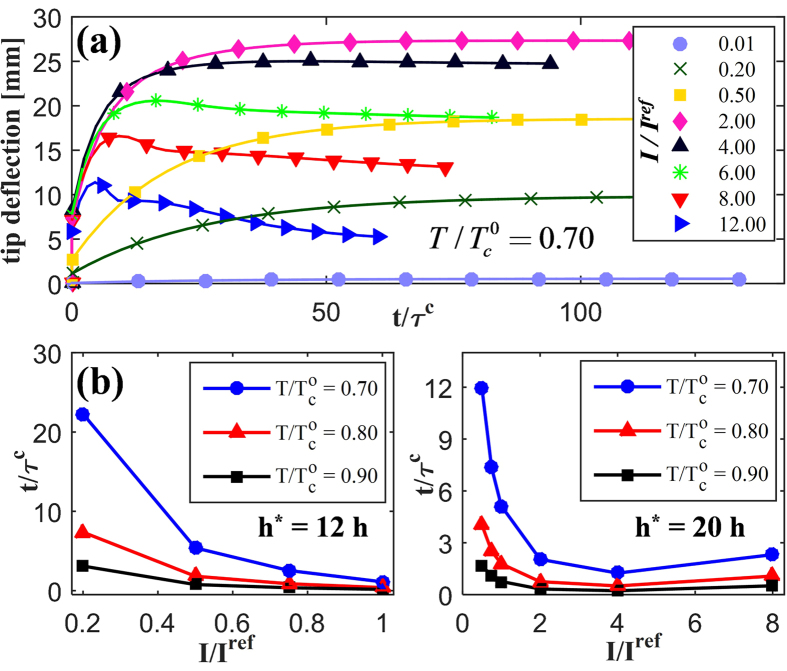
Time-dependent bending deformation of photo-responsive polymer networks (PRPNs). (**a**) Tip deflections compared with various light intensities *I/I*^*ref*^, wherein nonlinearities such as bending-unbending behaviour^35^ are found. (**b**) Characteristic time required to obtain specific deflections (h*) with different operating temperatures, wherein the thermal relaxation effect with the photo-bleaching effect is demonstrated. (h represents the thickness of the strip).

The overall behaviour of the PRPN is summarized in Fig. 3. Figure 3(a) illustrates the tip deflection (z-position) of the PRPN with respect to time; here we can clearly observe non-monotonic deflection. The saturated value of the final deflection increases with intensity for low-intensity light conditions (i.e. 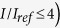, though it decreases as the intensity increases beyond this threshold, as the shape parameter profile suggests. Furthermore, as shown for 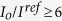, the reduced deformations do not settle and continue to decrease even beyond the time constant 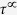.

At a point near the base (i.e. point A in Fig. 2), the accumulated high *cis-* population significantly dilutes the mesogenic order, and hence renders the local polymeric structure isotropic. The tip of the PRPN is subsequently lowered because of the flattened base, which decreases the incident angle *ϕ*, and hence the local equilibrium is attained again. At this stage, the deflection is then further decreased until the PRPN becomes totally flat^35^. The time required to obtain a specific tip deflection (h^*^ = 16 *h* or 20 *h*) is examined in Fig. 3(b). A decaying profile can be observed^33^ as the characteristic time becomes more sensitive to light for lower intensities. The relaxation of the polymeric segment due to increased temperature is also reflected in the shift of the curves for different values of *T*. This behaviour provides insight into the PRPN’s experimentally observed characteristic bending phenomena^33^,^35^, which could not be explained solely based on *a priori* estimations based on photo-strain^43^,^44^.

**Figure 4:**
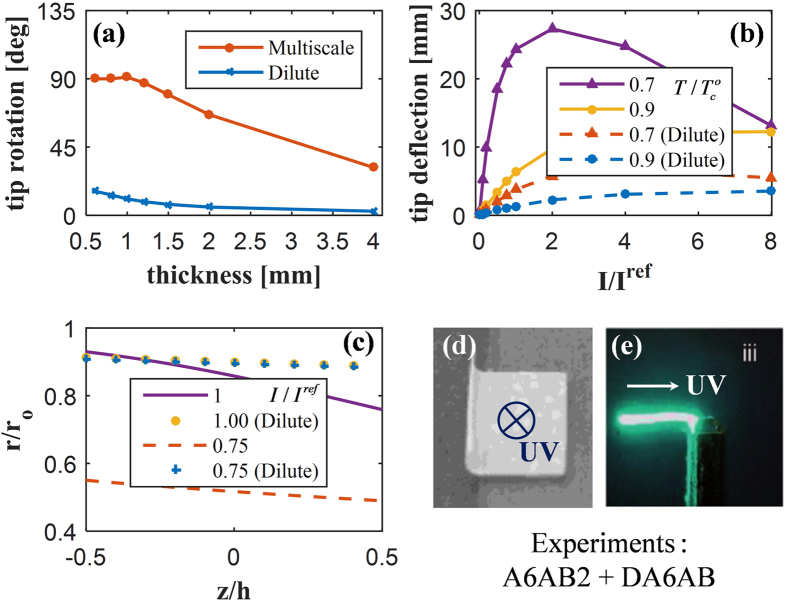
Bending deformation of photo-responsive polymer network (PRPN) computed via multiscale framework compared to the classical dilute model suggested by Hogan *et al*.^39^ Substantial differences are found in both (**a**) tip rotations and (**b**) tip deflections with various values of 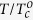. (**c**) With different light intensities, thickness gradients of the shape parameter 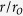 of the multiscale model (lines) are compared to those of the classical model (dots) in order to explain these differences. A 90° tip rotation is commonly observed in experiments of side-chain acrylate PRPNs whenever irradiated (**d**) from above or (**e**) from the left. Figure (**d**) is adapted with permission from Yu *et al.*^34^ (DOI: 10.1021/cm035092g). Copyright 2004 American Chemical Society. Figure (**e**) is adapted from White *et al.*^37^ (DOI: 10.1039/B805434G) with permission of Royal Society of Chemistry.

In order to exemplify the benefits of the methodology presented in this work, we also compare the results obtained by the present framework to those from the classical dilute model, as shown in Fig. 4. All classical results present hereafter (marked as “Dilute”) are obtained using the previous finite element study^31^ with constant thermotropic parameters (*α* = 1.4, *ζ* = 0.33, and 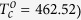 through MD simulations under non-irradiation conditions (*n*_*cis*_ = 0). Additionally, the order-clearing temperature is assumed to decrease linearly according to the following formula: 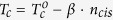, where *β* = 88.8. In both computations, 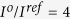, 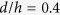, and rotations and displacements are obtained at the tip at time 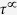.

First, a rotation is computed across a wide range of PRPN thicknesses (Fig. 4(a)). A deflection of 90° for the tip is expected for thin sheets; such a configuration is widely observed in experiments (see Fig. 4(d,e)) concerning side-chain acrylate PRPNs^33^,^35^,^37^. As Fig. 4(a) illustrates, the multiscale framework provides a better estimation of the shape change over the other solution. In particular, the deflection of the tip computed by the classical dilute model is significantly underestimated; the deflection remains far below 90° even when the bending stiffness is significantly reduced owing to the high length-to-thickness ratio (>70). Furthermore, the results of tip displacement (Fig. 4(b)) similarly indicate that the classical solution underestimates the shape change.

Such deviation can be attributed to the slope of the reduced anisotropy *r*/*r*_*o*_ curve, as illustrated in Fig. 4(c). The two models are utilized for the case of identical geometry, light intensity, and temperature; accordingly, they yield the same 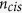
distributions. A stronger gradient of *r* in the multiscale model is therefore induced by a decrease/increase in *α*/*ζ*, as reflected in Fig. 1(b). It encompasses an additional effect from *trans-to-cis* isomerization that affects the order itself; the classical results, insofar as they do not account for the stacking characteristic, therefore underestimate the deformations.

It is worth mentioning that such effects from polymeric architecture can also be found in the silicon-based side-chain liquid crystal elastomer^3^ when it incorporates crosslinked-type azobenzene (aside from the pendant variety). More specifically, it undergoes order change with an increasing number of *cis-* isomers. This similarity clearly demonstrates that the multiscale simulation and concomitant lower-scale *in silico* experiment are capable of accounting for the molecular conformation in general opto-mechanical structures. However, the effects of changes in the polymeric structure upon the mechanical properties, such as the optimum actuation temperature and light-induced stress, cannot be validated in the present work, as we only provide preliminary considerations of the effects on bending and nonlinear photomechanics. It is therefore necessary to consider temperature-induced molecular flexibility (considered separately from order-induced change) in future studies.

## Methods

The multiscale framework presented in this work, which consists of micro-scale simulations and a scale-bridging method, incorporates information on microstate evolution and macro-scale deformation. Below we present a detailed description of the framework, although we omit certain equations and simulation conditions that can be found in the references^23^,^31^.

### Phase transition and concomitant behaviour of acrylate side-chain PRPN

We model the molecular unit cell of the acrylate-based PRPN, a polymeric structure with many mesogenic constituents, following the cell construction scheme suggested by Choi *et al.*^23^, which incorporates energetic relaxation and multi-step crosslinking. The molar crosslinking ratio is set to 7:1^45^, and energetic interactions and equilibrated polymeric conformations are computed via a photoactive potential^23^,^24^,^46^. The dihedral parameters of the azodye, as well as the potential coefficients of peripheral atoms, surrogate those of the conventional polymer consistent force field (PCFF). The simulations are performed with the program Large-scale Atomic/Molecular Massively Parallel Simulator (LAMMPS; heating-up simulations for photomechanical effect evaluation) and are complemented by Material Studios (crosslinking and cell synthesis) from Accelrys.

**Figure 5:**
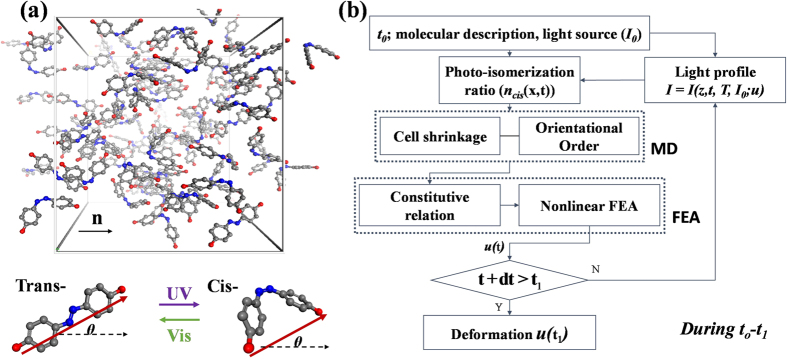
(**a**) Unit cell of a photo-responsive polymer network (PRPN) with *cis-*population 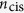
= 0.25 (flexible hydrocarbon networks are removed for readability) and *trans-* and *cis-* state molecules with angular deviation (*θ*) from the nematic director 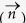. (**b**) Multiscale schematics. A photoisomerization ratio at time *t* is computed for a given light intensity 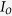 and temperature *T*; it is then used for molecular dynamics (MD) simulations, which provide microscopic information to the nonlinear finite element analysis (FEA).

A unit cell of a partially isomerized PRPN system with a temperature of 300 K is shown in Fig. 5(a), wherein dihedral angles of azodye molecules (-C-N=N-C-) indicate the transition between *trans-* (180°) and *cis-* (0°) states. The initial alignments of the chromophores are indicated by the director vector 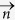. We observed that azodye molecules reorient vibrantly upon isomerization and heating-up simulations, which possibly changes the molecular configuration and of their networks; this is reflected in the thermodynamic behaviour of PRPNs parameterized by an orientational order parameter (*S*) and a cell shrinkage parameter (*λ*), which vary with increasing temperature. The orientational order parameter characterizes the long-range symmetry of mesogenic alignment; it ranges from 0 (no directionality) to 1 (perfect alignment), and is computed as an ensemble-averaged parameter (see Eq. (3)) that is a function of the mesogen-director deviation angle *θ*.


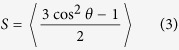


The shrinkage parameter *λ* is defined as the fractional change in length from the beginning of the simulation (300 K) to the point at which the order-clearing temperature 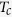 is reached. Even though 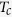 is defined as the point at which *S* becomes zero, we neglect the data after *S* is reduced to 0.05, as shown in Fig. 1(a); it highly fluctuates around 0 (i.e. the moving average of *S* settles to 0) as each mesogen is free to vibrate as the mesogenic symmetry is completely lost. Parametric fitting for the thermotropic phase transition parameters *α*/*ζ* is carried out in the temperature range of 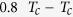 in order to elucidate the phase behaviour near this transition.

The accurate estimation of the order parameters and the shrinkage determines the overall results of this work. MD simulations are therefore executed many times by changing the initial setup, such as the position of the *cis*- isomers, the temperature ramping-up speed, etc. As these changes significantly burden the computational load, we store and reuse the results of the thermotropic parameters by fitting in a piecewise manner in order to consider locally varying the 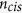 value distributed on the PRPN structure. Although not shown here, changing the fitting scheme to a 3^rd^-order polynomial does not change the results, insofar as they continue to vary monotonically.

### Scale-bridging approach toward photomechanics

The information flow and computations involved are described in Fig. 5(b). The multiscale framework is adopted in order to upscale the microstate changes found in MD simulations; these are then used to simulate macro-scale deformation subjected to a specified molecular composition (here, the acrylate side-chain PRPN), temperature *T*, and intensity of the light source 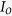.

First, 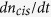 is determined as a function of *I*, the absorption coefficient *η*, and *T* with thermal relaxation ratio 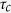, as shown in Eq. 42,3,26,30. Unlike the temperature, which takes on a homogeneous value throughout the PRPN strip, the light distribution is computed locally for each element according to the formula 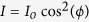, where the incident angle *ϕ* is the defined as the angle between each incident ray and the normal vector, which constantly changes owing to deformation. According to this formula, incident angles other than 90° decrease the absorbed number of photons^33^. We assume that the penetration depth *d* and profile of light are instantaneously equilibrated^47^ (relative to the speed of the 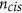 evolution). The Lambert W function is adopted to approximate the nonlinear optical absorption and concomitant nonlinear light penetration; a photo-bleaching effect^15^,^16^,^17^,^18^,^19^,^20^,^21^ that changes both the temporal and spatial distributions of *cis-* concentration is thereby considered. The implicit Euler method with a time-step of 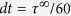 is used for numerical time integration, in which 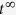 indicates the approximate settling time computed by Eq. (5). A 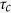 is computed in Eq. (6), assuming that the thermal-induced *trans-to-cis* isomerization follows Arrhenius-like behaviour for given activation energy Δ.


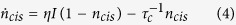



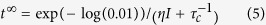



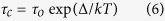


Next, a shape parameter *r* (which is equal to *λ*^3^)^31^ is computed to describe the microstate, as shown in Fig. 1(b). We assume that metric shape tensors of the polymer are degenerated to a single parameter *r*, as polymeric conformation remains uniaxial before and during photomechanical deformation^27^,^48^. That is because the MD simulation^23^ results show that neither biaxiality nor compressibility is provoked during the spontaneous deformation of MD unit cells.

A macroscopic bending deformation for a given microstructural distribution is computed quasi-statically^31^ for given time step *t*, in which phase behaviour is coupled to a geometric nonlinear shell in order to compute large-scale deformation. The incident angle *ϕ* for every element is computed consecutively for a given deformation and rotation, which in turn changes the light intensity and thus the 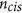 distribution on the surface. The loop continues until *t* reaches 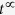. The simulation parameters are summarized in Table 2.

The present upscaling scheme, which uses shape parameter *r* as a bridging value, is therefore understood as a sequential method that is suitable for obtaining a quasi-static solution, as well as distinguishing the timescales of separate physical domains. These assumptions, however, are often violated if the question is related to dynamics such as rapid vibration^37^, wherein mechanical energy transfer is strongly coupled to microstate evolution.

## Conclusion

In summary, we present an analysis of the photomechanical behaviour of PRPNs through the newly developed multiscale framework. By incorporating the micro-scale behaviour of the polymeric network into the finite element simulation of liquid crystal networks, the light-driven manipulation of the PRPN sheet can be numerically explored across a wide range of operating temperatures and light intensities. Through these MD simulations, we consider the effects of the concentration of *cis-* molecules on the intrinsic phase behaviours of PRPNs: in particular, the linear relation between the order parameter *S* and the shrinkage parameter *λ*, and the roughly 2^nd^-order phase change near the clearing temperature. These two characteristics are shown to be retained but modified as the *cis-* population increases; the related parameters 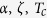 show monotonic changes upon increases in 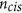, which suggests that mesogenic stacking influences the structural deformation, in addition to the classical dilution effect on the mesogenic order. Such differences are reflected on the macro-scale via the upscaling method, which considers the effects of the shape parameter upon the finite element framework^31^. Furthermore, the characteristic bending behaviour of the acrylate-based PRPN is simulated and agrees well with experimental values, thereby demonstrating the improved accuracy of the simulation. On the other hand, substantial differences are found in comparison with the classical dilute model, which underestimates the deformation owing to a lack of consideration of the polymeric composition in smaller scales.

It is worth noting that the present work rests on the assumptions made regarding the photoisomerization (i.e. Eqs. (4)–(6), 38, 39). We simplify the interactions between photons, temperature, and azodye with a simple ordinary differential equation, which offers much room for improvement via experiment and quantum simulations^10^,^49^. Our results should therefore be understood in terms of microstates in polymers and their effect on the deformation behaviour. Furthermore, downscaled behaviours, such as stress-induced soft elasticity and biaxiality^1^,^50^,^51^, should be considered in order to achieve a more comprehensive multiscale analysis.

## Additional Information

**How to cite this article**: Chung, H. *et al.* Nonlinear photomechanics of nematic networks: upscaling microscopic behaviour to macroscopic deformation. *Sci. Rep.* **6**, 20026; doi: 10.1038/srep20026 (2016).

## Figures and Tables

**Table 1:** Molecular dynamics (MD)-based microstate parameters.

*n*_*cis*_	*T*_*c*_ [K] (S.D.)	*α* (S.D.)	*ζ* (S.D.)
0.00	462.50	1.40	0.33
0.25	425.17 (33.32)	1.00 (0.10)	0.37 (0.03)
0.51	392.50 (9.70)	0.92 (0.25)	0.39 (0.06)
0.75	373.70 (7.52)	0.91 (0.33)	0.40 (0.13)
1.00	363.01	0.90	0.40
0^34^	443.15	N.A.	N.A.
0^39^	340.15	0.51	0.19

Standard deviation (S.D.) is calculated for cells with different locations of *cis-* molecules. References include thermodynamic properties from the acrylate side-chain^34^ and elastomer-based nematic polymer^39^.

**Table 2:** Simulation parameters; *μ* is the shear modulus.

Value [Units]		Value [Units]	
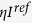 , 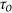	0.01, 25.3 [s^−1^]	Length × Width × Height	36 × 4 × 0.5 [mm]
Δ^39^	4 × 10^−20^ [J]	*d/h*	0.4
*μ*^35^	1.5 [GPa]	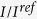	0.1–15
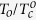	0.70	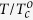	0.70, 0.80, 0.90
